# Low cost automated whole smear microscopy screening system for detection of acid fast bacilli

**DOI:** 10.1371/journal.pone.0190988

**Published:** 2018-01-22

**Authors:** Yan Nei Law, Hanbin Jian, Norman W. S. Lo, Margaret Ip, Mia Mei Yuk Chan, Kai Man Kam, Xiaohua Wu

**Affiliations:** 1 Hong Kong Applied Science & Technology Research Institute Co., Ltd. (ASTRI), Hong Kong SAR, China; 2 Department of Microbiology, Faculty of Medicine, The Chinese University of Hong Kong, Hong Kong SAR, China; 3 Stanley Ho Centre for Emerging Infectious Diseases, Faculty of Medicine, The Chinese University of Hong Kong, Hong Kong SAR, China; Indian Institute of Technology Delhi, INDIA

## Abstract

**Background:**

In countries with high tuberculosis (TB) burden, there is urgent need for rapid, large-scale screening to detect smear-positive patients. We developed a computer-aided whole smear screening system that focuses in real-time, captures images and provides diagnostic grading, for both bright-field and fluorescence microscopy for detection of acid-fast-bacilli (AFB) from respiratory specimens.

**Objectives:**

To evaluate the performance of dual-mode screening system in AFB diagnostic algorithms on concentrated smears with auramine O (AO) staining, as well as direct smears with AO and Ziehl-Neelsen (ZN) staining, using mycobacterial culture results as gold standard.

**Methods:**

Adult patient sputum samples requesting for *M*. *tuberculosis* cultures were divided into three batches for staining: direct AO-stained, direct ZN-stained and concentrated smears AO-stained. All slides were graded by an experienced microscopist, in parallel with the automated whole smear screening system. Sensitivity and specificity of a TB diagnostic algorithm in using the screening system alone, and in combination with a microscopist, were evaluated.

**Results:**

Of 488 direct AO-stained smears, 228 were culture positive. These yielded a sensitivity of 81.6% and specificity of 74.2%. Of 334 direct smears with ZN staining, 142 were culture positive, which gave a sensitivity of 70.4% and specificity of 76.6%. Of 505 concentrated smears with AO staining, 250 were culture positive, giving a sensitivity of 86.4% and specificity of 71.0%. To further improve performance, machine grading was confirmed by manual smear grading when the number of AFBs detected fell within an uncertainty range. These combined results gave significant improvement in specificity (AO-direct:85.4%; ZN-direct:85.4%; AO-concentrated:92.5%) and slight improvement in sensitivity while requiring only limited manual workload.

**Conclusion:**

Our system achieved high sensitivity without substantially compromising specificity when compared to culture results. Significant improvement in specificity was obtained when uncertain results were confirmed by manual smear grading. This approach had potential to substantially reduce workload of microscopists in high burden countries.

## Introduction

Global tuberculosis (TB) burden is still enormous even after years of efforts to reduce its incidence and mortality. According to the World Health Organization (WHO) 2017 report [1], 6.3 million new cases of TB were reported in 2016 (up from 6.1 million in 2015) equivalent to 61% of the estimated incidence of 10.4 million, and 1.7 million deaths resulted from TB in 2016 globally.

Most of the high incidences were in developing countries and areas where diagnostic instrumentation and medical professionals were in severe shortage. With today’s globalization trend, TB infection poses a threat not only to high incidence countries, but also to the whole world. It is therefore urgent to provide quick and effective diagnostic solutions for TB. Secondly, current microbiological diagnostic methods of TB still require improvement. Culture diagnosis remains the gold standard but it takes weeks to obtain results, while smear microscopy provides quick diagnosis but with limited sensitivity.

Current sensitivity of manual smear microscopy diagnosis is highly variable, with sometimes up to almost half of acid fast bacilli (AFB) positive smears being mistakenly graded as negative [2]. As a consequence, true TB patients remained undetected by smear microscopy and no anti-TB treatment can be started before culture results are available. Not only would the patient not be given the proper anti-TB treatment, but this also meant no effective control measures could be in place to prevent spread of infection to others, whether in hospital or in the community. In some situations, the low sensitivity was mainly due to failure of detecting “scanty” positive smears with very few AFBs, when only about 10% of the smear area (2 cm×1cm under 200x) was examined using commonly recommended practice [3]. Although new technologies such as the Xpert MTB/RIF (GeneXpert) molecular tests help improve reliability in detection of TB, they are not easily affordable to many low- to middle-income countries. Despite its shortcomings, smear microscopy continues to be the most widely used tool for TB diagnosis in high-burden developing countries.

In the smear microscopy screening process, AFBs are searched in either auramine-O (AO) stained or Ziehl-Neelsen (ZN) stained smears under fluorescence or bright-field microscope respectively. Fluorescence microscopy, due to its higher contrast and larger field-of-view (FOV), has been used to screen smears for higher throughput. However, objects other than AFB can also emit green fluorescence under excitation, which may cause false-positive results. Therefore, smears diagnosed as positive in fluorescence microscopy are usually confirmed by re-checking the slide using ZN bright field microscopy at higher magnifications. In Hong Kong, this two-step screening process was often adopted by TB laboratories to balance the work throughput and diagnostic accuracy. Therefore, smear screening tool development for these two modes were critical and equally important.

To effectively reduce human error while keeping our objective of low-cost, we developed a computer-aided AFB whole-smear microscopy screening system which included real-time focusing, digitally capturing images, as well as providing diagnostic grading based on the number of AFBs identified by computer vision algorithms. Our system worked in both bright field and fluorescence microscopy modes.

Previous research works related to this important area have been published [4, 5, 6]. Most of these only proposed solutions for particular components, however, without getting together into one holistic system. There had been two automated microscopy systems for this application [7]. The first was the TBDx system from Signature Mapping Medical Science Inc. [8]. Its performance when combined with manual microscopy [9] and use of GXP as a confirmatory test [10] had been described. However, the evaluation was only done on concentrated-smears using AO-staining. The second was an automated smear microscopy reader from Becton Dickinson. This was under development and no further detailed information was available. Another system was known as Fluorobot [11], but no detailed description and system performance could be found in the published literature.

Our study aimed to examine the performance of a dual-mode screening system in AFB diagnostic algorithms on concentrated smears with AO staining, as well as direct smears with AO and ZN staining. The evaluation was conducted and analysed using results produced by our screening system alone, as well as in combination with a microscopist, with AFB culture results as the gold standard. Comparison of machine grading with manual smear grading was also made.

## Materials and methods

### Ethics statement

Ethical approval for this study was obtained from the Joint Chinese University of Hong Kong–New Territories East Cluster Clinical Research Ethics Committee.

(http://www.crec.cuhk.edu.hk/)

Respiratory samples were collected and saved from patients suspected of clinical respiratory infections, with requests for mycobacterial cultures and AFB smears prepared for subsequent staining and examination.

### Sample preparation

Sputum smears were prepared according to standard diagnostic microbiology laboratory protocols. Each sample was smeared over a 2cm^2^ area on a clean glass slide. For AO staining, smears were air dried and fixed by gentle heating. Each slide was then stained with 0.1% auramine phenol (Merck, Darmstadt, Germany), decolorized in 0.5% acid-alcohol (0.5% conc. HCl in 70% ethanol), counterstained with 0.5% potassium permanganate (Merck, Darmstadt, Germany), and then air-dried. Smears were examined by experienced microscopists covering at least 30 microscopic fields for typical slender fluorescent rods under fluorescence microscope using 250x magnification. For ZN staining, smears were air-dried and fixed by gentle heating. Each slide was then flooded with 1.2% carbol fuchsin (Merck, Darmstadt, Germany), decolorized in 3% acid-alcohol, counterstained with dilute malachite green solution 0.5%, and rinsed with water. Slides were examined for at least 300 fields under light microscope at 1000x magnification for typical AFB slender rods. To prepare concentrated specimens, sputum digestion-decontamination with equal volume of N-acetyl-L-cysteine-NaOH (making final volume of approximately 3 mL) and centrifugation (3000g for 15 min.) were done before staining. Following the two-step screening process mentioned in the Introduction Section, the grading based on the WHO guideline was given by an experienced microscopist who worked for years in a routine TB diagnostic laboratory.

### AFB screening

The smears were then scanned and graded using the automated scanning platform, blinded to all other test results. Slides were freshly stained with auramine-O before scanning to ensure that objects appeared bright and sharp in the digitized images, as seen under routine diagnostic laboratory conditions.

The automated system consisted of a modified Motic motorized microscope platform that enabled both fluorescence and brightfield mode whole smear scanning. After a sputum smear sample was loaded, a low magnification whole slide preview image (with identification barcode) was acquired by scanning with a 2x/0.05 Numerical Aperture (N.A.) objective lens, and the sputum smear microscopy area (typically a 2cm by 1cm ellipse area) was automatically selected from the whole smear image. Then high magnification images were acquired from the selected smear microscopy area by scanning with a 20x/0.5N.A. objective lens, with fluorescence images for AO stained smears under blue LED excitation (through an optical filter cube) and brightfield images for ZN stained smears under white LED illumination. The imaging camera was a Point Grey 5MP color camera with 2/3 inches CCD sensor. The pixel resolution for the captured image was 0.17 micron, with the exposure time of 70ms and 2ms for fluorescence and brightfield imaging respectively. Each FOV size was determined by the CCD camera sensor size and the objective lens magnification. Thus, the total number of FOVs covered on the selected smear microscopy area could be derived (with a tiny overlap between neighboring FOVs). With a proprietary real-time auto-focusing module, it took less than 5min to complete one smear scan under both imaging modes.

The image processing and analysis algorithm for both fluorescence and brightfield smear microscopy FOV images were carried out during the scan, thus the AFB detection results were available immediately after the smear microscopy scan was completed. For fluorescence images, only morphology and brightness features were utilized to segment out the AFB candidates and separate them into AFB or non-AFB categories with a classifier based on both SVM and Decision Tree. For brightfield images, color information was also taken into account to design the feature vector to conduct AFB segmentation and classification. Each FOV of the slide was then analyzed and the maximum number of AFBs detected in one optical field length was returned for further diagnostic grading. The detected AFB numbers from all the FOVs were then added together to determine the total number of AFBs of a smear. To be consistent with the WHO TB smear grading criteria, the number of AFBs was calculated for each “length”, i.e. a whole strip of consecutive FOVs along the long axis of the ellipse smear microscopy area. The smear then was automatically graded into one of the five levels (negative, scanty, 1+, 2+, 3+) based on the grading standards for TB diagnosis (3), depending on the total number of AFB detected as shown in Table 1. The critical AFB threshold to separate “Negative” and “Scanty” was tuned to balance sensitivity and specificity. After repeated determinations in the training step, these were set at 9 and 6 AFBs for fluorescence and brightfield modes respectively.

**Table 1:** 5-level grading scheme for AFB slides.

	Grading standards from (Lumb, Deun, Bastian, & Fitz-Gerald, 2013) (1 field in 1000x ≈ 0.035mm^2^)	Machine grading scheme (1 length ≈ 7mm^2^) (*δ* = 9 for AO-stained, *δ* = 6 for ZN-stained)
Negative	No AFB in 1 length	<*δ* AFB in 1 length
Scanty	1–9 AFB in 100 fields	δ-20 AFB in 1 length
1+	10–99 AFB in 100 fields	20–200 AFB in 1 length
2+	1–10 AFB per field	200–2000 AFB in 1 length
3+	> 10 AFB per field	>2000 AFB in length

### Validation

Two analyses were performed to evaluate our dual-mode screening system in TB diagnostic algorithms, viz.: results produced by our screening system alone, and then results in combination with a microscopist. The workload of independent microscopist for each setting was also reported.

The final mycobacterial culture results were used as the gold standard throughout all experiments. Two statistical performance measurements of our system were reported: sensitivity was defined as the proportion of culture positive cases which were machine graded as positive; specificity was defined as the proportion of culture negative cases graded as negative.

Finally, the comparison of machine grading and manual smear grading was also made. Overall performance of TB diagnostic algorithms was assessed by the percentage symmetric difference between two positive-negative grading results defined as the proportion of disagreement sets, and the major discrepancy (more than one grade difference) rate between two 5-level grading (negative, scanty, 1+, 2+, 3+) results.

## Results

**Fig 1:**
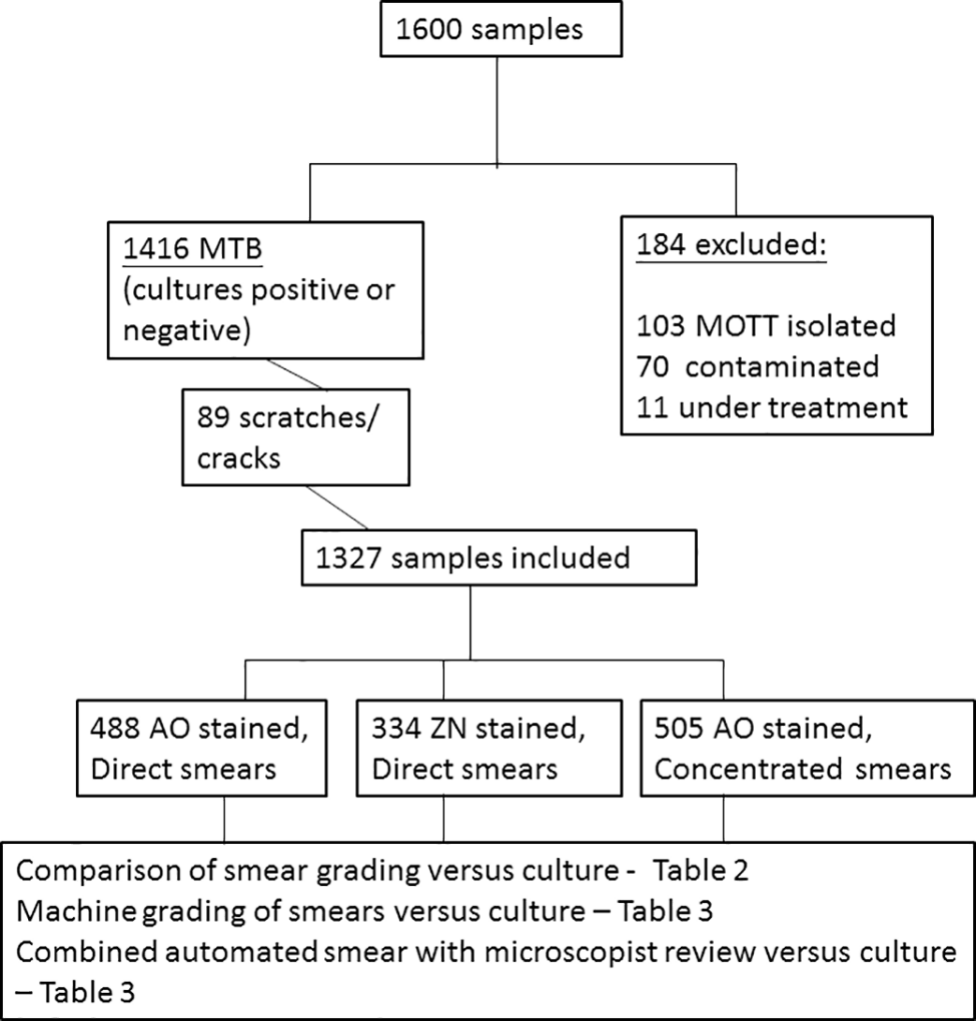
Flow chart of sample processing.

A total of 1600 sputum samples from adult patients with respiratory illness and requesting for Ziehl-Neelsen staining and mycobacteria cultures were included. Of all specimens, 1416 were either culture positive for *M*. *tuberculosis* or culture negative, 103 were culture positive for *Mycobacteria* spp. other than *M*. *tuberculosis* complex (MOTT), 70 were overgrown with contaminants, and 11 were from treated cases. A flow chart of sample processing is shown in Fig 1.

Specimens were divided into three batches for evaluation. From these smears; 89 contained scratches or cracks rendering inability of the system to auto-focus, and were excluded from evaluation. The ratio of positive to negative samples was about 1-to-1.

### Comparisons of smear grading vs culture

Of 488 direct smears with AO-staining, 228 were culture positive. There were 203 smears graded as positive, giving a sensitivity of 89.0% [95% CI: 84.2–92.8%] (Table 2). The remaining 260 were culture negative, of which 250 were graded as smear negative, giving a specificity of 96.2% [95%CI: 93.8–98.5%]. Out of 334 direct smears with ZN staining, 142 were culture positive. There were 122 graded as smear positive, resulting in a sensitivity of 85.9% [95%CI: 79.1–91.2%]. Of the remaining 192 that were culture negative, 181 were graded as smear-negative, giving a specificity of 94.3% [95%CI: 90.0–97.1%]. For 505 concentrated smears with AO staining, 250 were culture positive, and 220 of these were graded as smear positive, resulting in a sensitivity of 88.0% [95%CI: 83.3–91.8%]. The remaining 255 were culture negative, all of which were graded as smear negative, giving a specificity of 1 [95%CI: 98.6–100%].

**Table 2:** The sensitivity and specificity of microscopist’s smear grading, compared to culture results and the corresponding microscopist’s workload for the three batch categories.

Type of smear and staining	Sensitivity [95%CI]	Specificity [95%CI]	No. of slides reviewed by microscopist
AO Direct	89.0 [84.2–92.8]	96.2 [93.8–98.5]	488
ZN Direct	85.9 [79.1–91.2]	94.3 [90.0–97.1]	334
AO Concentrated	88.0 [83.3–91.8]	100 [98.6–100]	505

### Machine grading of smears versus culture

Using the automated system for reading and grading the smears of AO stained direct smears, 186 were graded as machine positive (i.e., in which the system detected at least 9 AFBs) which were culture positive, giving a sensitivity of 81.6% [95%CI: 75.9–86.4%] (Table 3). There were 193 smears graded as machine negative were culture negative, giving a specificity of 74.2% [95%CI: 68.5–79.4%]. For the batch of ZN stained direct smears, 100 smears were graded as machine positive (i.e. in which the system detected at least 6 AFBs) were culture positive, giving a sensitivity of 70.4% [95%CI: 62.2–77.8%]. There were 147 smears graded as machine negative that were culture negative, giving a specificity of 76.6% [95%CI: 69.9–82.4%]. For the batch of AO stained concentrated smears, 216 smears graded as machine positive were culture positive, giving a sensitivity of 86.4% [95%CI: 81.5–90.4%]. On the other hand, 181 graded as machine negative were culture negative, giving a specificity of 71.0% [95%CI: 65.0–76.5%].

**Table 3:** The sensitivity and specificity of TB diagnostic algorithms, compared to culture results and the corresponding microscopist’s workload for the three batches.

Batch	Sensitivity [95%CI]	Specificity [95%CI]	No. of slides reviewed by microscopist
**(1) Stand-alone by automated system**			
AO Direct	81.6 [75.9–86.4]	74.2 [68.5–79.4]	0
ZN Direct	70.4 [62.2–77.8]	76.6 [69.9–82.4]	0
AO Concentrated	86.4 [81.5–90.4]	71.0 [65.0–76.5]	0
**(2) Confirmed by smear grading**			
AO Direct	86.0 [81.5–90.5]	85.4 [81.1–89.7]	131
ZN Direct	77.5 [70.6–84.3]	85.4 [80.4–90.4]	70
AO Concentrated	87.2 [83.1–91.3]	92.5 [89.3–95.8]	148

### Combined automated smear with microscopist review versus culture

**Fig 2:**
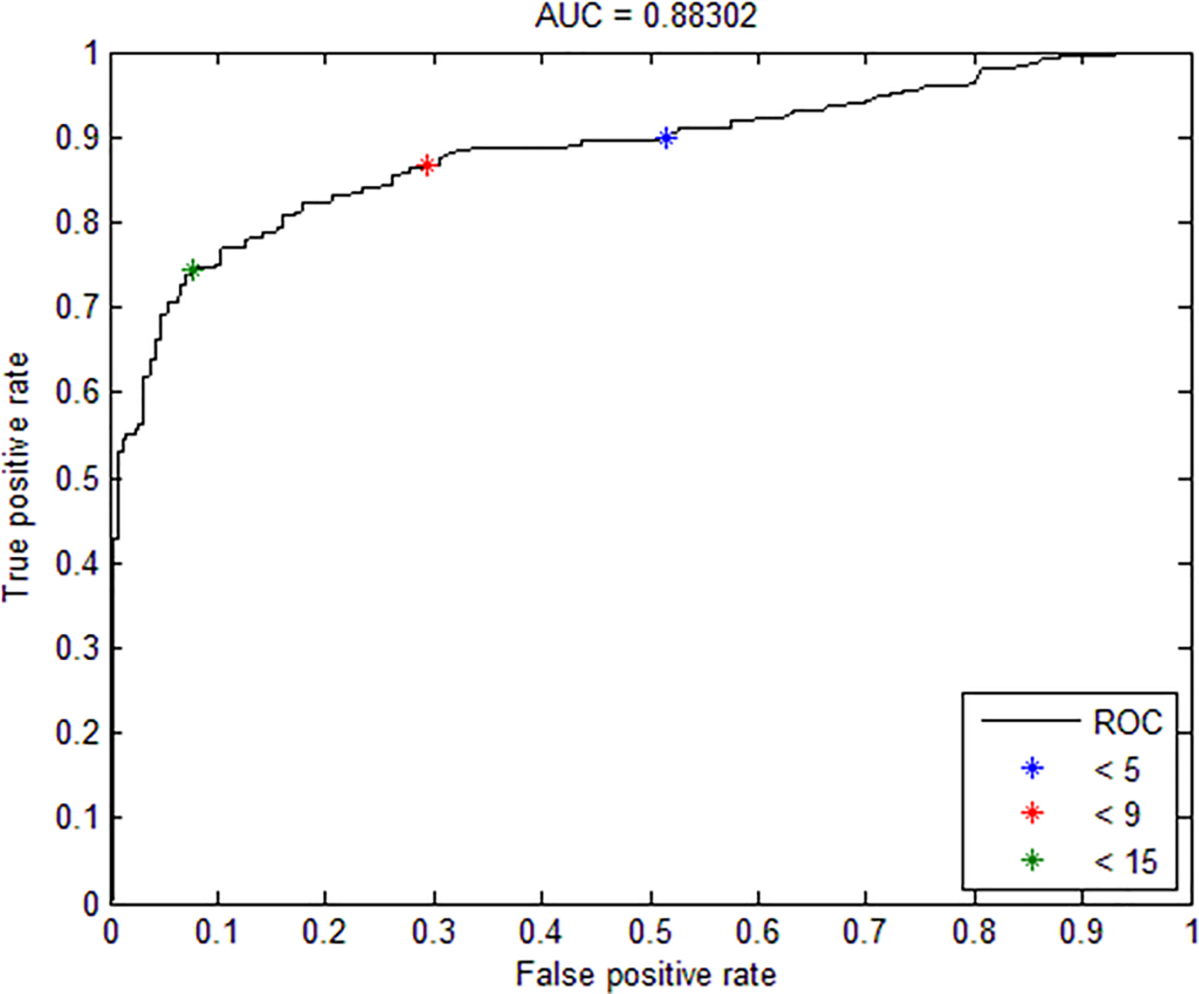
Receiver operating characteristic curve showing the trade-off between sensitivity and specificity of the diagnostic algorithm as the grading cutoff changes for the AO stained concentrated smear microscopy batch.

For the two AO stained batches, a good sensitivity and fair specificities were obtained by machine-reading; while the reverse was obtained for the ZN stained batch. In order to improve the overall performance of the results while minimizing any additional manual workload, an algorithm was designed by adjusting the cutoff number of positively-stained bacilli during machine grading. The limits of detection of the number of AFB cutoffs were set for a negative smear (i.e. five for AO and three for ZN) and a positive smear of bacteria (‘scanty’ grade) (i.e. 15 for AO and 13 for ZN) respectively. Fig 2 shows the trade-off between sensitivity and specificity of the diagnostic algorithm as the grading cutoff changes for the AO stained concentrated batch. In particular, the sensitivity increased to 89.6% when the cutoff for a positive smear was set to be > 5 (i.e., black asterisk in Fig 2), as a result of dropping specificity to 49.8%. On the other hand, the specificity could be increased to 92.5% when the cutoff was set at > 15 (i.e. green asterisk in Fig 2), as a result of dropping the sensitivity to 74.4%. Note that this range gave a much improved performance in terms of better specificity while keeping good sensitivity. The results obtained by combining automated smear with microscopist review gave a significant improvement in specificity while at the same time a slight increase in sensitivity. As shown in Table 3 for the batch of AO-stained direct smears, 196 graded as smear-positive were culture positive, giving a sensitivity of 86.0% [95%CI: 81.5–90.5%] (Table 3). There were 222 graded as smear-negative that also turned out to be culture negative, giving a specificity of 85.4% [95%CI: 81.1–89.7%]. For the batch of ZN stained direct smears, 110 were graded as positive were proven culture positive, giving a sensitivity of 77.5% [95%CI: 70.6–84.3%]. There were 164 graded as negative were culture negative, giving a specificity of 85.4% [95%CI: 80.4–90.4%]. The corresponding improvement in performance for AO-stained concentrated smears showed sensitivity reaching 87.2% [95%CI: 83.1–91.3%], and specificity 92.5% [95%CI: 89.3–95.8%].

### Comparison of the machine grade scores versus smear grading by microscopist

The performance of the system in the TB diagnostic algorithm was assessed by comparison of the smear scores performed by the microscopist. The scores of AO stained direct smears are listed in Table 4. The percentage symmetric difference was 16.0% (Table 4) and the major discrepancy rate was 12.5%. For ZN stained direct smears, the percentage symmetric difference was 17.4% (Table 5) and the major discrepancy rate was 8.4%. For AO stained concentrated smears, the percentage symmetric difference was 15.4% (Table 6) and the major discrepancy rate was 9.7%.

**Table 4:** The matching matrix between results of smear and machine 5-level grading for AO stained direct smears.

Number of AFBs detected by system	Smear negative	Smear positive			
		Scanty	1+	2+	3+
0–9	216	8	10	1	0
10–20	32	13	4	3	0
21–200	27	30	41	12	9
201–2000	0	6	24	13	17
>2000	0	0	5	3	14

**Table 5:** The matching matrix between results of smear and machine 5-level grading for ZN stained direct smear batch.

Number of AFBs detected by system	Smear negative	Smear positive			
		Scanty	1+	2+	3+
0–6	166	19	4	0	0
7–20	26	15	16	3	0
21–200	9	8	17	14	10
201–2000	0	0	8	2	11
>2000	0	0	2	0	4

**Table 6:** The matching matrix between results of smear and machine 5-level grading for AO stained concentrated smear batch.

Number of AFBs detected by system	Smear negative	Smear positive			
		Scanty	1+	2+	3+
0–9	210	1	2	0	0
10–20	66	20	22	15	0
21–200	9	9	15	47	21
201–2000	0	2	1	21	26
>2000	0	0	0	2	16

## Discussion

To the best of our knowledge in the searchable literature, this is the first description of a comprehensive evaluation of an automated whole-smear microscopy scanning system for detection of AFB on sputum smears using a combination of AO and ZN staining. The advantage of having whole smear scanning was to avoid false negatives due to some AFBs being excluded from the scanning. However, the bottleneck of such a system was the time required to scan a whole smear. Our system was designed to overcome this by using large FOV and by speeding up the auto-focusing as well as AFBs recognition steps. The whole scanning and analyzing processes took only approximately 5 minutes for a 2cm×2cm smear region. Our system achieved a high sensitivity without substantially compromising specificity, when compared to mycobacterial culture results, and showed high consistency even with successive smear grading from scanty to 3+. To further improve the performance, the machine grading was confirmed by the smear grading when the number of AFBs detected by the system fell in a range with uncertain values. This approach only required 27% of specimens to be examined by the microscopist while obtaining a significant improvement in specificity. We noted that the proportion of the specimens requiring confirmation was slightly highly than those described in previous studies [9, 10]. This might be due to the fact that the positive-negative ratio of the samples used in this study was different from those in other studies.

Another advantage of having whole smear scanning was that the system provided a finer grading (negative, scanty, 1+, 2+, 3+) which were based on the distribution of the detected AFBs in the whole smear. According to published smear examination guideline [3], the microscopist should first scan the whole smear in low power and then confirm the suspicious objects using high power. Then the specimen should be graded based on the number of identified AFBs. Based on the guideline, there may not be sufficient information for giving finer grading when only partial smears were examined.

Most of the previous studies focused on automatic AFB detection from images obtained by only one mode of microscopy. In this study, we examined the performance of our dual-mode screening system in TB diagnostic algorithms using concentrated smears with AO-staining, as well as direct smears with AO- and ZN-staining. We proposed that smear screening tools for different modes were equally important for high-burden countries setting. The system achieved the highest sensitivity on the batch of AO-stained concentrated smears, which was consistent with results obtained in [12] which stated that the sensitivity could be greatly increased by concentration technique. The sensitivity we achieved was higher than those reported in another study [10]. Amongst the three batch categories used, the system achieved the highest specificity but the lowest sensitivity on the batch of ZN-stained direct smears. It might be due to difficulties in detection of AFBs under bright field microscopy: 1) lower sensitivity when compared to fluorescence microscopy [13]and 2) calibration required due to color variation of carbol fuchsin dye in specimens from different stain batches [6]. Overall, our dual-mode screening system performed well in all these three different settings.

In TB high-burden low-income countries, it is important to keep the system cost at affordable range. To achieve this while maintaining good performance, we adopted a mid-range microscope and computer. Together with the TB diagnostic kit, our system cost was approximately USD20,000. Compared to other platforms such as TBDx[8] which costs USD23,000 for both components (software license not included) [7], our solution was more definitely attractive. In comparison with other reported automated TB smear diagnosis-support systems (e.g. TBDx from Signature Mapping), our system was designed with a low cost motorized microscopy system. In contrast to the Olympus microscope, optics, camera and Prior Scientific slide loader, our Motic system and Point Grey camera were much less costly. Moreover, our system had the following advantages: firstly, the system could be easily configured to operate in either fluorescence or brightfield mode. The only change that needed to be done was to switch the light source and filter cube. Secondly, with our proprietary auto focusing module, the system could complete a whole smear microscopy scan within 5min which covered more than 1200 FOVs under a 20x objective lens, while other systems could deal with much less numbers (<100 FOVs) within the same time frame. Thirdly, we utilized LED light sources instead of the Mercury lamp used in traditional fluorescence microscopes or those used in traditional brightfield microscopes, with a significant benefit for lower cost as well as longer lifetime.

One potential limitation of our system was that currently the slides were manually placed on the stage for scanning. The system could, however, be easily configured to operate with a mechanized slide loader or with an automated stage with multiple slide feeding capacity. In terms of performance, the sensitivity of our present system had been substantially increased by whole smear microscopy scanning approach without much compromise on specificity. On the other hand, specificity was greatly improved by confirming those showing uncertain machine-generated grading with a subsequent proper smear grading. In situations where laboratory expertise might be limited, another confirmatory test such as Xpert MTB/RIF tests could be considered as well. In future, the performance of detection algorithms should be optimized further to eliminate remaining false-positive smears.
